# Exploratory Study of CD10^low^ Polymorphonuclear Leukocytes Preceding and Correlating With Postsurgical Inflammation

**DOI:** 10.1111/sji.70042

**Published:** 2025-07-13

**Authors:** Timo Michael Westermann, Joe Sendas, Alexander Sebastian Koller, Darko Jovanovski, Dominik Hüsken, Pascal Max Lucien Lessing, Birte Weber, Bernd Mühling, Andreas Liebold, David Alexander Christian Messerer, Markus Huber‐Lang, Lisa Wohlgemuth

**Affiliations:** ^1^ Institute of Clinical and Experimental Trauma Immunology University Hospital Ulm Ulm Germany; ^2^ Department of Cardiothoracic and Vascular Surgery University Hospital Ulm Ulm Germany; ^3^ Department of Trauma Surgery and Orthopaedics Goethe University Frankfurt Frankfurt Germany; ^4^ Institute for Transfusion Medicine University Hospital Ulm Ulm Germany; ^5^ Institute for Clinical Transfusion Medicine and Immunogenetics German Red Cross Blood Transfusion Service Baden‐Württemberg‐Hessen and University Hospital Ulm Ulm Germany

## Abstract

The lack of diagnostic and monitoring tools for postsurgical immunological complications such as systemic inflammation can contribute to a poor outcome despite modern intensive care. The need for reliable immune monitoring has been emphasised. Polymorphonuclear leukocytes (PMNs) play an important role in postsurgical inflammation. A subgroup of PMNs is particularly interesting, because they are released shortly after (iatrogenic) trauma: immature PMNs, characterised by, for example, their low CD10 expression. Therefore, we investigated the role of CD10^low^ PMNs in a non‐interventional exploratory study by including patients undergoing scheduled, highly standardised cardiac surgery with extracorporeal circulation. We were able to demonstrate that the number of CD10^low^ PMNs released shortly after the beginning of surgery correlated with different fluid phase markers of inflammation and organ damage postsurgically. Among these parameters were CRP, IL‐6, NGAL, CK‐MB, and troponin‐T. Noteworthy, the amount of CD10^low^ PMNs increased as early as 24 h before these well‐established markers, suggesting superiority of CD10^low^ PMNs as an early diagnostic marker. Comparing CD10^low^ immature PMNs with CD10^high^ mature PMNs revealed potential involved mechanisms, including lower CD11b expression and a significant decrease in the formation of platelet–neutrophil complexes (PNCs) by CD10^low^ PMNs. In conclusion, we propose CD10^low^ PMNs as a potential early cellular biomarker to assess the postsurgical inflammatory response. In comparison to clinically established markers like CRP or IL‐6 and scoring systems such as the SOFA‐Score, CD10^low^ PMNs reflect a potential candidate for future immune monitoring to determine the risk of excessive inflammation and organ impairment more rapidly.

## Graphical abstract

The present exploratory study aimed to assess whether cellular immune monitoring could be an option to detect the postsurgical inflammatory response in patients undergoing cardiac surgery of aortic valve replacement accompanied with extracorporeal circulation. Particular attention was given to CD10^low^ polymorphonuclear leukocyte (PMN), which showed a marked increase already during surgery. Notably, the rise of CD10low PMNs preceded the elevation of classical systemic inflammation markers such as C‐reactive protein (CRP), interleukin‐6 (IL‐6) and procalcitonin (PCT). These findings raise the possibility that CD10low PMNs could serve as an early cellular marker for identifying patients at risk of developing inflammatory postsurgical complications.

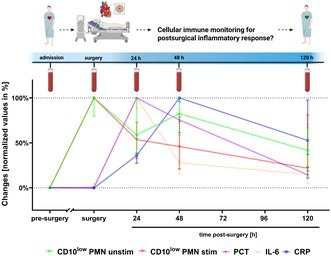

## 1 Introduction

As increasing numbers of individuals survive the acute phase of trauma [1], posttraumatic and postsurgical immunologically‐driven complications become increasingly relevant [2]. The disruption of physiologic barriers and the release of damage‐associated molecular patterns (DAMPs) trigger systemic inflammation. Systemic inflammatory response syndrome (SIRS) and complications (e.g., pneumonia, sepsis) as well as hypoxic factors (e.g., haemorrhage, shock) frequently lead to multiorgan dysfunction associated with a high mortality rate [2, 3, 4, 5].

Although the importance of the immune system in the development and progression of posttraumatic and postsurgical complications is evident, the underlying mechanisms are not fully understood [2, 4, 6]. During the early posttraumatic phase, both inflammatory and anti‐inflammatory processes occur simultaneously [2, 7, 8, 9, 10, 11]. The immune system's response is mainly triggered by DAMPs, among them mitochondrial DNA, adenosine triphosphate and histones [8, 11, 12, 13]. However, the initiated inflammation can become overwhelming and progress to a systemic immune dysfunction in the form of SIRS [2, 7, 14]. For practising clinicians, the current lack of early diagnostic tools and targeted treatment options is still an unmet clinical need [2, 7]. Established inflammatory parameters (e.g., C‐reactive protein (CRP) and interleukins) and clinical scores, including the sequential organ failure assessment score (SOFA), frequently show delayed discriminative alterations and are, furthermore, quite unspecific [15, 16]. These circumstances can lead to the delay of important diagnostic and therapeutic steps, affecting patient outcome. In addition to fluid phase inflammatory markers, the need for monitoring of the cellular phase of the immune system by cellular immune monitoring has been proposed [7, 17, 18]. A potential candidate for this monitoring approach are polymorphonuclear leukocytes (PMNs) [17, 18, 19, 20], frequently described as the first line of innate cellular defence [21].

PMNs can be activated by DAMPs and complement activation products [22] and sense such danger signals, for example via complement and toll‐like receptors on their surface [23]. This activation leads to phenotypic changes (e.g., increased CD11b expression), reactive oxygen species (ROS) generation, and neutrophil extracellular trap (NET) formation [9, 11, 24]. Upon stimulation, PMNs may also form platelet–neutrophil complexes (PNCs) and alter their own phagocytic activity [9, 25, 26]. On the one hand, these processes are important to limit inside‐out bleeding and outside‐in bacterial migration. On the other hand, they can damage protective barriers and the host [27, 28]. The wide spectrum of PMNs functions has inspired extensive research suggesting that PMNs are divisible into many different subgroups [19, 20, 29, 30, 31, 32, 33]. Among these subtypes are immature PMNs, which upon a traumatic stimulus (i.e., trauma vector) are released into the bloodstream [31, 32]. These immature PMNs have different characteristics compared to their mature counterparts. For example, they exhibit an enhanced antibacterial activity [29]. To detect these immature PMNs by flow cytometry, different markers can be used, such as CD16, CD11b and CD10 expression [34, 35, 36, 37, 38]. CD10 (neprilysin) is of particular interest because it is only expressed in the final stages of PMNs maturation [35, 36, 37]. However, the exact role of these immature CD10^low^ PMNs in inflammatory complications remains unclear [38, 39, 40, 41, 42, 43]. Nevertheless, this subtype has been proposed for a potential immune monitoring approach [18, 19, 20, 40].

In the present exploratory study, we included patients undergoing cardiac surgery with the help of extracorporeal circulation covering the opportunity to investigate trauma‐related pathophysiologies prior, during, and after the injury. Within this setting, DAMPs are released during surgery [44, 45] and SIRS is a common postsurgical complication [45, 46, 47]. We investigated the relevance, features, and potential of CD10^low^ PMNs as a diagnostic, monitoring, or prognostic marker of inflammatory complications in patients undergoing cardiac surgery accompanied by extracorporeal circulation.

## 2 Materials and Methods

All chemicals were purchased from Sigma‐Aldrich (St. Louis, Missouri, USA), when not indicated otherwise. All antibodies and isotypes were purchased from BioLegend (San Diego, California, USA).

### 2.1 Patient Cohort and Sample Collection

The patient cohort was part of a prospective non‐interventional study with no clinical trial registry and a follow‐up cohort of a recently published study [48]. After ethical approval by the Local Independent Ethics Committee of the University of Ulm (452/21) and obtaining written informed consent, samples were collected from the patients. Patients were at least 18 years old, had no major comorbidities for the scope of the study (Table S1) and were scheduled for upcoming aortic valve replacement surgery with extracorporeal circulation under general anaesthesia (Table S2). Patients with revision procedures, active malignant diseases or undergoing immunomodulating treatment (e.g., chemo‐ or radiotherapy and immunosuppressants) were excluded. A total volume of 9 mL blood was drawn into syringes containing 3.2% trisodium citrate (#04.1902.001, Sarstedt, Nürnbrecht, Germany) and 1 mL into syringes containing 25 IU/mL lithium heparin gel (#04.1927.001 Sarstedt) on admission day before surgery (~24 h before), during surgery (from the venous branch 45 min after the initiation of extracorporeal circulation), and 24, 48 and 120 h after surgery (±10% time buffer within each measurement time point). Additionally, data from routine monitoring and routine blood drawing were collected (the corresponding measurements were conducted by the Clinical Chemistry Department of the University Hospital Ulm).

### 2.2 Determination and Measurement of the PMN Phenotypes

Samples with 10 μL whole blood per condition were either stimulated (stim) with a proinflammatory cocktail or phosphate‐buffered saline (PBS) containing calcium and magnesium (PBS^++^, #14040–091, Thermo Fisher Scientific, Darmstadt, Germany) as an unstimulated (unstim) control. The proinflammatory cocktail for the stim condition contained 10 μM N‐formyl‐Met‐Leu‐Phe (fMLF, #F3506, Sigma Aldrich, Steinheim, Germany), 1 μM platelet‐activating‐factor (PAF, #18779, Cayman Chemical, Ann Arbor, USA), and 2.3 μM human tumour necrosis factor (TNF, #570102, BioLegend). Samples were stained with different antibodies, including 4.0 μg/mL Alexa Fluor 700 anti‐human CD11b antibody (#301355), 4.0 μg/mL APC anti‐human CD184 antibody (#306510), 4.0 μg/mL FITC anti‐human CD66b antibody (#305104), 0.05 μg/mL PE anti‐human CD62L antibody (#304806) and 4.0 μg/mL PE/Cyanine7 anti‐human CD10 antibody (#312214). Corresponding isotype controls were used (CD11b: IgG1, k, #400144; CD184: IgG2a, k, #400220; CD66b: IgM, k, #401606; CD62L: IgG1, k, #400112; CD10: IgG1, k, #400126). After the addition of antibodies (unstim and stim), the samples were adjusted to a total volume of 50 μL using additional PBS^++^. These samples were incubated in a light‐protected water bath at 37°C for 15 min. Cells were fixed using 950 μL of 1× with distilled water diluted FACS Lysing Solution (BD Biosciences, San Jose, USA) for 30 min at room temperature in the dark. Subsequently, the samples were centrifuged at 340 ***g*** for 5 min. The supernatant fluid was discarded, and the cell pellet was resuspended in 100 μL PBS without calcium or magnesium (PBS^−^, #14190–094, Thermo Fisher Scientific) containing 1% bovine serum albumin (#A8022, Sigma‐Aldrich). Samples were stored at 4°C in the dark and measured within 30 min.

### 2.3 Measurement of PNC Formation

PNC generation was determined using 0.5 μg/mL PerCP/Cyanine5.5 anti‐human CD61 antibody (#336418) and as a control its respective isotype control (IgG1, k, #400150) as previously described in detail by our group [49]. The steps were identical to those for determining PMN phenotype described above, except that the incubation was extended to 30 min instead of 15 min. Furthermore, lithium‐heparin anticoagulated blood was used instead of trisodium citrate anticoagulated.

### 2.4 Flow Cytometry and Gating Strategy

The samples were measured for 2 min at a medium flow rate using the BD FACSLyric Flow cytometer (BD Biosciences, Franklin Lakes, USA). Data was analysed using bflow (Version 0.1.6.9, Bertram Dietrich Thomaß, bflow project, www.bflow.science, Ulm, Germany). After exclusion of doublets using the forward scatter area (FSC‐A) and FSC‐height (FSC‐H), PMNs were gated using their characteristic FSC‐A and the side scatter area (SSC‐A) (Figure S1). Gating of the CD10^low^ PMNs was performed on the PMNs population using the pre‐surgery measurement time point as a reference. This reference was established separately for all patients and both ex vivo conditions. First, a splitter gate was used to separate CD10^low^ and CD10^high^ PMNs in pre‐surgery samples under the assumption that ≥ 99% of PMNs were mature and therefore CD10^high^. This splitter gate was subsequently applied to the surgery group samples and to the post‐surgery samples, revealing CD10^low^ populations as post‐insult changes. This is displayed step‐by‐step in Figure S2.

### 2.5 Humoral Analysis by LEGENDplex Analysis

Trisodium citrate plasma samples were used to measure neutrophil gelatinase‐associated lipocalin (NGAL) using a commercially available LEGENDplex Kit (#740590, BioLegend) and performed as instructed by the manufacturer.

### 2.6 Statistical Analysis and Data Presentation

Results were presented as median ± interquartile range (IQR, 25th percentile–75th percentile), when not indicated otherwise. All data were considered to be unpaired and non‐parametric. Statistical significance was set at *p* < 0.05 with *, **, *** and ****, indicating < 0.05, < 0.01, < 0.001 and < 0.0001, respectively. Statistical analysis was conducted using GraphPad Prism 9 (GraphPad Software Inc., San Diego, California, USA) and Microsoft Excel 2019 (Microsoft Corporation, Redmond, Washington, USA). For correlation analysis, the Spearman correlation coefficient *r* was calculated unless otherwise indicated.

## 3 Results

### 3.1 Patients' Clinical Characteristics

A total of 12 patients (11:1 male: female) with a median age of 62.5 (IQR: 57.7–67.0) years were included in the presented study. To quantify organ dysfunction, the SOFA score for each patient was calculated at each measurement time point. The presence of inflammation was assumed at a SOFA score ≥ 2 (data not shown). Eleven out of 12 patients reached this threshold at least once during the first 120 h after surgery. The values at 24 h (4; IQR: 3–6) and 48 h (3; IQR: 1–4) were significantly higher (*p* < 0.0001 and *p* < 0.001 respectively, *n* = 12) than the pre‐surgery control time point of each patient. A detailed overview of the patient cohort is shown in Table S1. Additional relevant anaesthesia, surgical and perioperative data is listed in Table S2.

### 3.2 CD10^low^ PMNs Were Elevated During and After Surgery and Superior in the Early Detection of Inflammation

**FIGURE 1:**
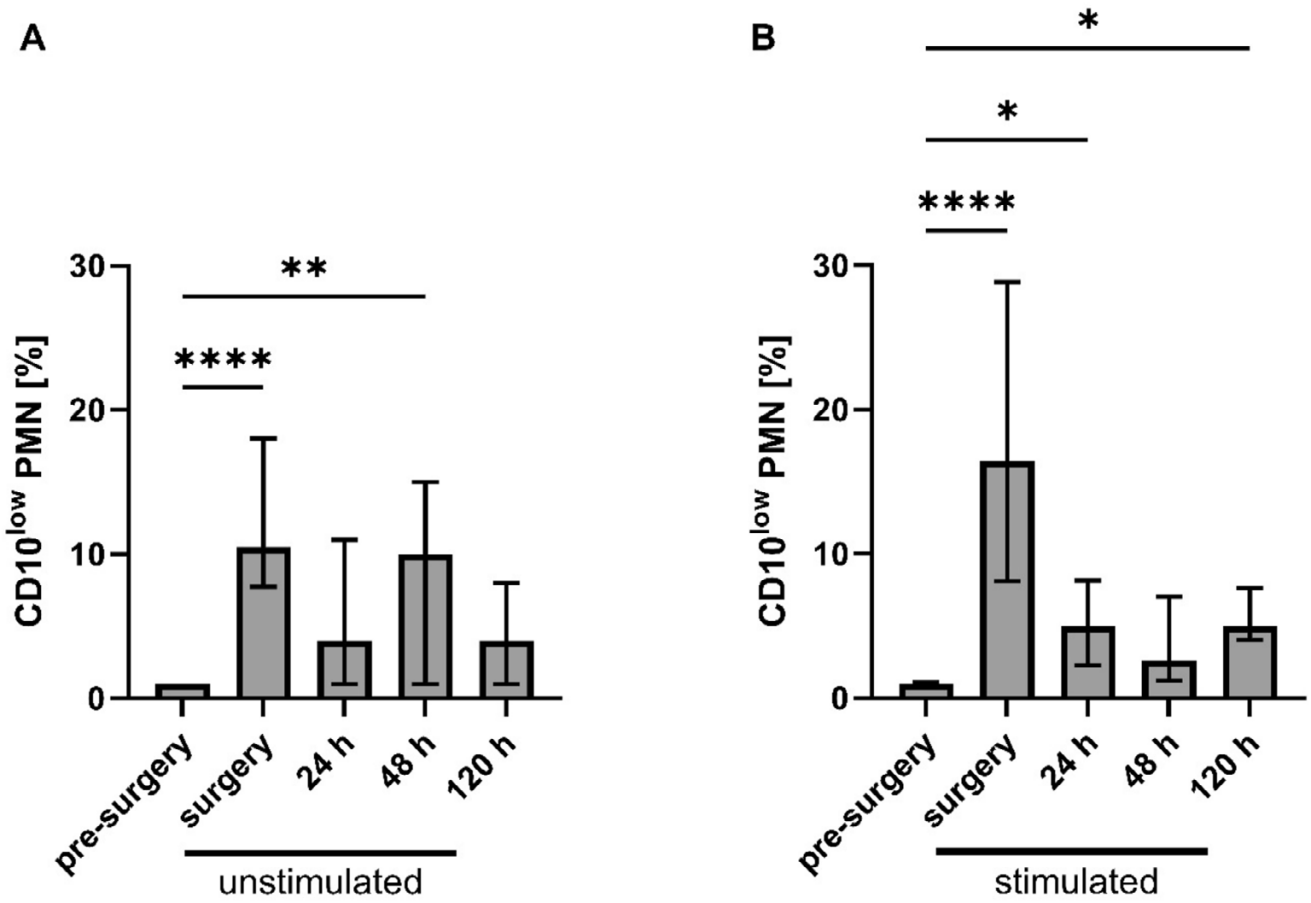
Perioperative numbers of CD10^low^ polymorphonuclear leukocytes (PMNs) in comparison to the total PMNs count. (A) CD10^low^ PMNs of total PMNs under unstimulated conditions before surgery (pre‐surgery), during surgery and 24, 48 and 120 h after surgery. (B) CD10^low^ PMNs numbers of total PMNs stimulated with cocktail (consisting of 10 μM fMLF, 1 μM PAF and 2.3 μM TNF) before surgery, during and 24, 48 and 120 h after surgery. Median ± interquartile range, *n* = 10. Kruskal–Wallis test with Dunn's post hoc correction when comparing to pre‐surgery with *p < 0.05, **p < 0.01, ****p < 0.0001.

The CD10^low^ PMNs appeared regardless of unstim conditions (Figure 1A) or stim conditions (Figure 1B) during and after surgery. During surgery, CD10^low^ PMNs represented 10.5% when unstim (IQR: 7.8%–18.0%; *p* < 0.0001 compared to pre‐surgery) and 16.5% when stim (IQR: 8.1%–28.9%; *p* < 0.0001 compared to pre‐surgery) of total PMNs. Additionally, the number of CD10^low^ PMNs was significantly elevated 48 h after surgery under unstim conditions (*p* < 0.01) and 24 h (*p* < 0.05) as well as 120 h (*p* < 0.05) after surgery under stim conditions (Figure 1). Attempts to generate a CD10^low^ population in an in vitro setting using proinflammatory mediators and DAMPs (lipopolysaccharides, histones, interleukin‐1β, PAF, fMLF, TNF) failed to induce the formation of CD10^low^ PMNs (data not shown), further supporting the concept that CD10^low^ PMNs are an in vivo phenomenon.

**FIGURE 2:**
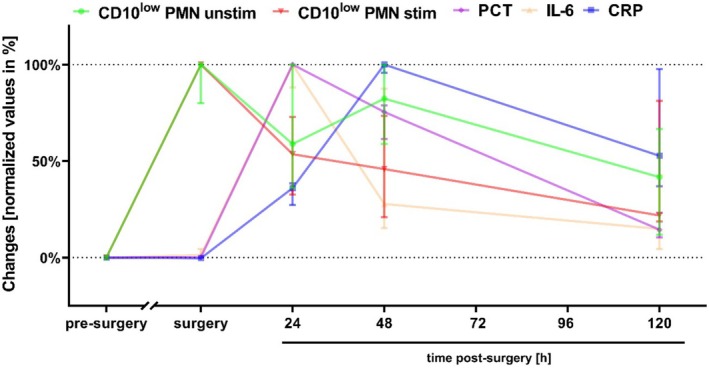
Comparison of CD10^low^ polymorphonuclear leukocytes (PMNs) numbers perioperatively without stimulation (unstim) as well as with stimulation by an inflammatory cocktail (stim). (the cocktail consisted of10 μM fMLF, 1 μM PAF and 2.3 μM TNF). CD10^low^ PMNs are compared with established clinical diagnostic markers of inflammation like procalcitonin (PCT), interleukin‐6 (IL‐6) and C‐reactive protein (CRP) before, during, and 24, 48, and 120 h after surgery. Data normalised to 0% = pre‐surgery and 100% = maximal change. Median ± interquartile range, *n* = 10.

The presence of unstim as well as stim CD10^low^ PMNs occurred before established clinical diagnostic markers for inflammation (CRP, interleukin‐6 (IL‐6) and procalcitonin (PCT)) increased significantly (Figure 2).

### 3.3 CD10^low^ PMNs During Surgery Correlated With Established Diagnostic Markers of Inflammation and Organ Damage

**FIGURE 3:**
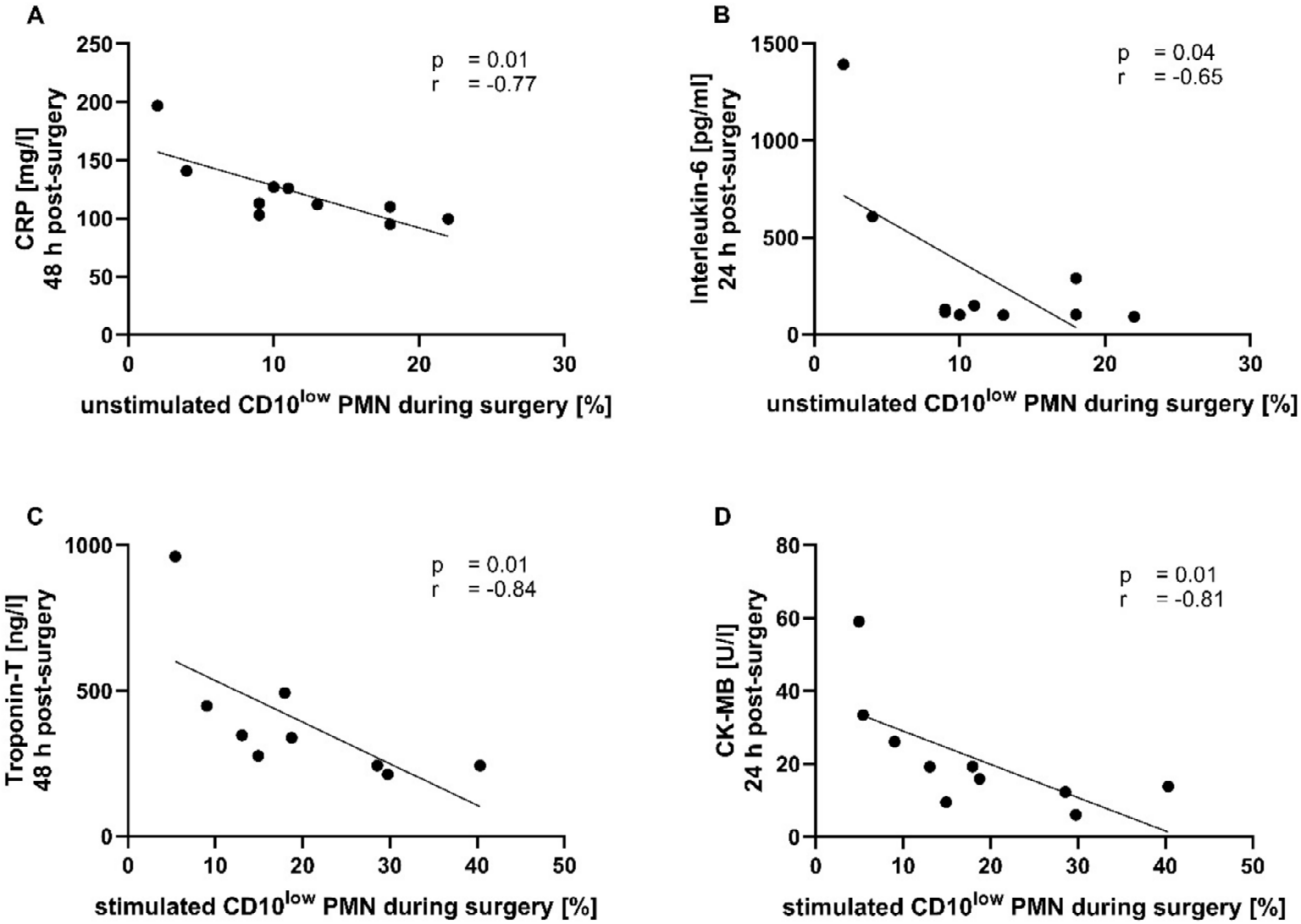
Simple linear regressions of CD10^low^ polymorphonuclear leukocytes (PMNs) of total PMNs during surgery and makers of inflammation and organ damage post‐surgery. (A, B) CD10^low^ PMNs under unstimulated conditions correlated with C‐reactive protein (CRP) at 48 h (A) and interleukin‐6 at 24 h (B) after surgery. (C, D) CD10^low^ PMNs under stimulation with an inflammatory cocktail (consisting of 10 μM fMLF, 1 μM PAF and 2.3 μM TNF) with troponin‐T at 48 h (C) and creatine kinase‐MB (CK‐MB) at 24 h (D). Spearman‐*r* correlation, *n* = 9–10.

To investigate whether CD10^low^ PMNs can serve as diagnostic and/or prognostic markers, the number of CD10^low^ PMNs during surgery was correlated with the change of different inflammatory and organ damage markers in the post‐surgery period. Under unstimulated conditions, the amount of CD10^low^ PMNs during surgery correlated significantly with both CRP at 48 h (*p* < 0.05, *r* = −0.77) and IL‐6 at 24 h (*p* < 0.05, *r* = −0.65) post‐surgery (Figure 3A,B). Under stimulated conditions, the correlations with troponin‐T at 48 h (*p* < 0.01, *r* = −0.84) and creatine kinase MB (CK‐MB) at 24 h (*p* < 0.01, *r* = −0.81) after surgery were significant (Figure 3C,D). Other inflammatory markers (such as PCT) did not exhibit relevant correlations (Figure S3).

**FIGURE 4:**
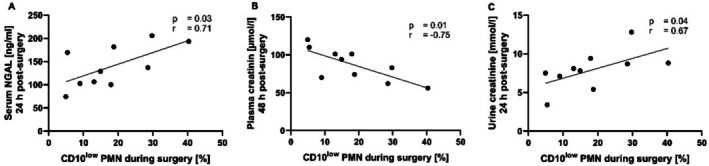
Simple linear regressions of stimulated CD10^low^ polymorphonuclear leukocytes (PMNs) of the total PMNs numbers during surgery and makers of kidney damage post‐surgery. (A) CD10^low^ PMNs under unstimulated conditions (with buffer as ctrl) and plasma neutrophil gelatinase‐associated lipocalin (NGAL) at 24 h, plasma creatinine at 48 h (B), and urine creatinine at 24 h (C) post‐surgery. Spearman‐*r* correlation, *n* = 10.

Liver damage markers (transaminases ALT and total bilirubin) did not correlate with the CD10^low^ PMNs numbers (Figure S3). By contrast, upon ex vivo stimulation, the number of CD10^low^ PMNs during surgery correlated significantly with plasma NGAL at 24 h (*p* < 0.05, *r* = 0.71), plasma creatinine at 48 h (*p* < 0.05, *r* = −0.75) and urine creatinine at 24 h (*p* < 0.05, *r* = 0.67) after surgery (Figure 4).

### 3.4 CD10^low^ PMNs Exhibit an Altered Phenotype and Decreased PNC Formation Compared to CD10^high^ PMNs

**FIGURE 5:**
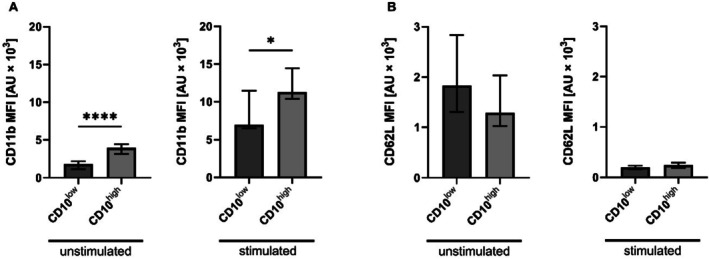
Comparison of surface antigen expression of CD10^low^ polymorphonuclear leukocytes (PMNs) and CD10^high^ PMNs during surgery under unstimulated conditions (with buffer as ctrl) and after stimulation with an inflammatory cocktail (consisting of 10 μM fMLF, 1 μM PAF and 2.3 μM TNF). (A) CD11b and (B) CD62L from patients during surgery. Median ± interquartile range, *n* = 10. Mann–Whitney U Test with *p < 0.05, ****p < 0.0001.

**FIGURE 6:**
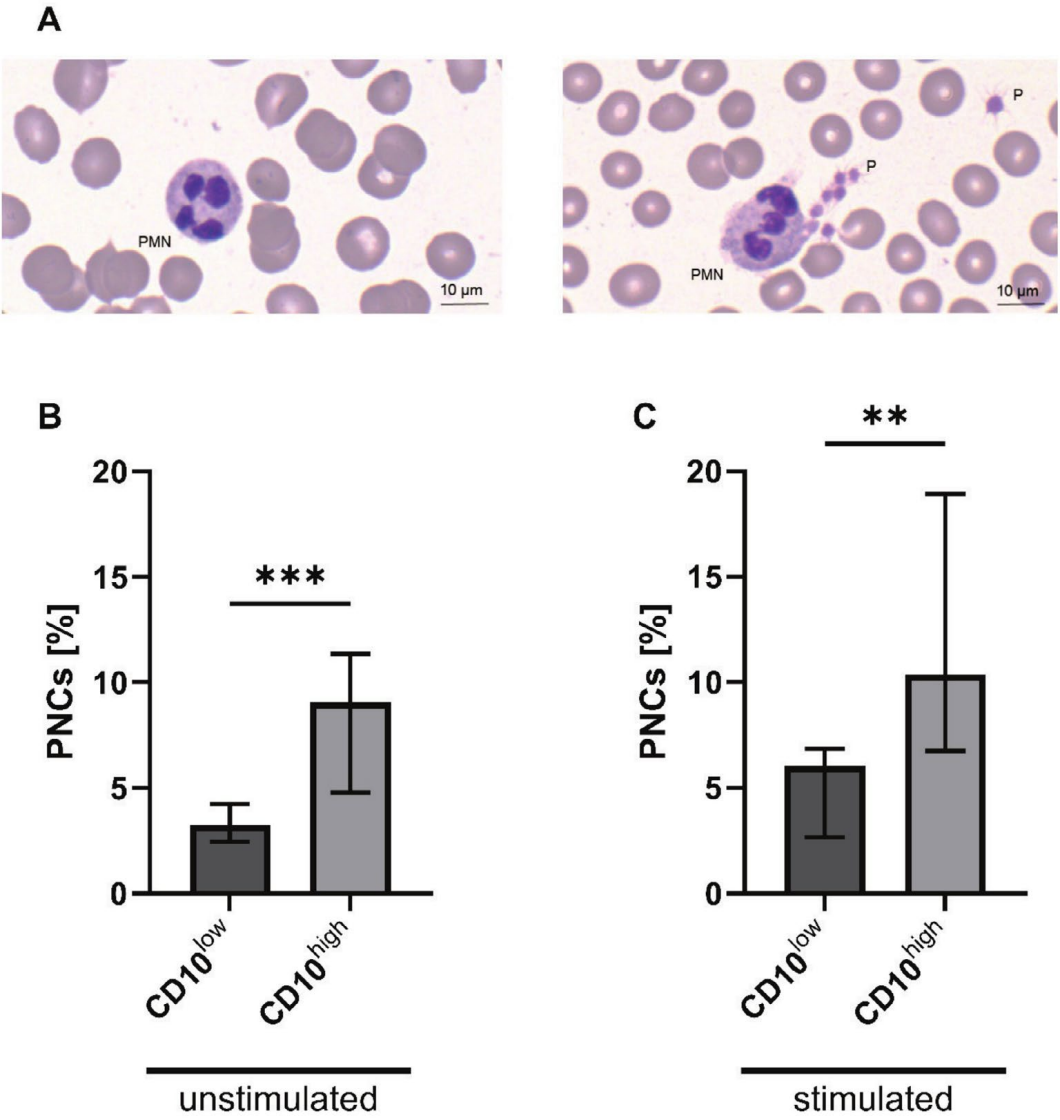
Formation of platelet–neutrophil complexes (PNCs) of CD10^low^ polymorphonuclear leukocytes (PMNs) and CD10^high^ PMNs during surgery. (A) Representative PNC as detected by light microscopy (right). Platelets are marked as (P). (B) Flow cytometric analysis of PNC formation under unstimulated conditions (with buffer as ctrl), and (C) stimulated conditions with an inflammatory cocktail (consisting of 10 μM fMLF, 1 μM PAF and 2.3 μM TNF). Median ± interquartile range, *n* = 10. Mann–Whitney U test with **p < 0.01, ***p < 0.001.

While the activation marker CD11b showed lower surface expression levels in CD10^low^ PMNs than in CD10^high^ PMNs (Figure 5A, unstim *p* < 0.0001, stim *p* < 0.05), the activation marker CD62L, which is shed upon activation, did not exhibit significant changes (Figure 5B). The CD66b and CD184 expression (Figure S4) was similar in CD10^low^ and CD10^high^ PMNs. Regarding the thromboinflammatory response, when focusing on the formation of PNCs, CD10^low^ PMNs formed significantly fewer complexes during surgery in comparison to the CD10^high^ PMNs (Figure 6, unstim *p* < 0.001, stim *p* < 0.01).

## 4 Discussion

### 4.1 CD10^low^ PMNs as Immature PMNs

Whether the CD10^low^ PMNs represented immature PMNs could not be definitively substantiated. Despite this, multiple indications support this assumption, including a morphological immaturity [39, 50]. Previous studies showed the release of CD10^low^ PMNs into the blood stream upon in vivo stimulation [35, 38, 51, 52]. The fact that attempts to replicate the generation of CD10^low^ PMNs by adding inflammatory stimuli in vitro did not result in increased CD10^low^ PMNs appearance underlines that they exclusively appear in vivo, as they migrate from the bone marrow into the blood stream [38]. Additionally, the combination of low CD10 and CD16 expression has been associated with immaturity [34, 35]. Furthermore, CD11b, which exhibited a reduced expression in CD10^low^ PMNs, can also be used as a marker of immaturity [34]. The similar expression of CD184 in CD10^low^ and CD10^high^ PMNs partially contradicts these findings because CD184 is expected to be expressed less in immature PMNs [53]. The fact that most of the identified correlations were observed under stimulated conditions is noteworthy. A potential explanation for this is that CD10 is partly stored in intracellular vesicles [54, 55, 56]. Upon stimulation, CD10 antigens are transferred to the cell surface. Therefore, under unstimulated conditions, some mature PMNs might be identified as CD10^low^ while still being able to express CD10 upon stimulation. Ex vivo stimulating the PMNs therefore might have increased the precision of CD10^low^ immature PMNs detection because only PMNs unable to express CD10 were labelled as definitive CD10^low^ PMNs. This theory cannot be verified by the existing literature because stimulation has not been used to increase accuracy in the other studies investigating CD10^low^ PMNs [40, 41, 42, 43].

### 4.2 CD10^low^ PMNs as an Early Indicator for Inflammation and Prognostic Parameter

Flow cytometry is well‐suited for clinical practice because of its time efficiency and the small sample volumes required. Modern cytometers can be used without additional laboratory equipment or trained personnel [57]. Furthermore, flow cytometry is also state‐of‐the‐art at differentiating PMNs into their heterogeneous phenotypes using surface antigens [20, 35]. PMNs have also previously been suggested as an approach to immune monitoring in posttraumatic and postsurgical inflammation [18, 19, 20, 40]. To assess inflammation, fluid phase markers such as CRP, IL6, PCT and the neutrophil/lymphocyte ratio (NLR) were selected due to their use in clinical practice (CRP and IL‐6) and current research (NLR) [58]. PCT was used as a marker of septic complications because of its high specificity for bacterial infection. In the present study, higher numbers of CD10^low^ PMNs were associated with reduced inflammation (lower CRP and IL‐6 levels) and less organ damage (lower troponin‐T, CK‐MB, creatinine and NGAL levels). A relationship between CD10^low^ PMNs and the cardio‐renal axis [59, 60] appears to be evident. This is further supported by previous results showing an association between CD10^low^ PMNs and cardiac diseases [61, 62]. The association between CD10^low^ PMNs and inflammation as well as organ damage demonstrates their suitability as prognostic parameters. This is further supported by the important fact that CD10^low^ PMN numbers increased much earlier, around 24 h before other clinically established markers indicating inflammation. Previous studies were able to link increased CD10^low^ PMN numbers to adverse outcomes in sepsis and postsurgical complications [40, 41, 42, 43]. CD10^low^ PMNs being associated with decreased inflammation and organ damage in this present study may appear to conflict with the previously observed adverse outcomes. However, inflammation is an ambivalent and highly time‐dependent phenomenon, and its prognostic value remains unclear [7]. Additionally, previous studies did not use a pre‐insult measurement time point as a reference and furthermore did not stimulate the blood samples in vitro. This could imply that the accuracy in CD10^low^ immature PMN detection was minor in comparison to the levels presented in the current study.

### 4.3 Potential Underlying Mechanisms

CD10^low^ PMNs expressed reduced levels of CD11b, which can be linked to their impaired ability of degranulation [63, 64]. Additionally, CD10^low^ PMNs formed fewer PNCs. PNC formation is important for extravasation and NETosis in PMNs [65, 66, 67]. This is supported by evidence that immature PMNs have been shown to exhibit a different NETosis behaviour than mature PMNs [68, 69]. These aspects could have multiple consequences for the organism. NETosis, in particular, was shown to be associated with increased PMNs auto‐aggressive potential [27]. Moreover, in the context of thromboinflammation, PNCs may also function as a cellular focal point for thrombi formation, which may be less possible in the case of the CD10^low^ PMNs phenotype. Immature PMNs have also been shown to stimulate lymphocyte survival and proliferation [38, 39]. Additionally, CD10^low^ PMNs increase IFN‐γ production by lymphocytes [38, 39]. This interferon has multiple immune system modulating functions and, in turn, affects PMNs [70, 71]. This IFN‐γ‐mediated association between CD10^low^ PMNs and lymphocytes plays an important role during sepsis and graft‐versus‐host diseases [39, 72]. Because lymphocytes are also pivotal in (iatrogenic) trauma, this mechanism could also be relevant [71, 73]. However, in the present study, no fluctuations in IFN‐γ levels were observed during or after surgery (data not shown). Overall, CD10^low^ PMNs may not necessarily be in a causal relationship with inflammation. In particular, they could be influenced by dysregulated recruiting signals during severe inflammation. PMNs recruitment is impaired after repeated in vivo stimulation with LPS [31] and could therefore explain the identified association between low numbers of CD10^low^ PMNs and higher levels of inflammation.

### 4.4 Strengths and Limitations

Using cardiac surgery as a standardised model for (iatrogenic) trauma and DAMP‐driven inflammation [45, 47] allowed us to obtain data in a strictly defined setting. Because most of the patients developed SIRS post‐surgery, we were able to investigate the CD10^low^ PMNs in both earlier and later phases of systemic inflammation. A key strength of this study is the identification of significant correlations between CD10^low^ PMNs and established markers of inflammation and organ damage. Furthermore, the additional in vitro stimulation with the inflammatory cocktail potentially increased the accuracy of CD10^low^ PMNs detection compared to previous studies [41, 42, 43]. The utilisation of pre‐surgery measurements could be considered both a strength and a limitation. Using pre‐insult data as an individual reference for each patient allowed precise differentiation of CD10^low^ PMNs appearing during and after surgery and the CD10^high^ PMNs that were already in circulation. Although this might be useful for diagnosing post‐surgery inflammatory complications, pre‐insult measurements are not feasible for trauma patients. Therefore, different methods of CD10^low^ PMNs detection will have to be applied to transfer these findings to trauma patients. A possible solution could be to use average numbers of healthy individuals as cut‐off values, as used in different methods of immature PMNs identification, using CD16 and CD62L signals [31]. The influence of heart disease on PMNs heterogeneity could have also influenced our findings [61, 62]. Quantification of inflammation using the SOFA score is common in clinical practice but has limitations. In this study, the SOFA score was used because of its validation as a screening tool with high sensitivity and because it covers more organ systems than other scores [74]. The small sample size of the patients that were included is a weakness of the study that also led to no subgroup analysis being performed. While correlations in such small cohorts are possible in principle, they nonetheless require validation through studies involving larger patient numbers. Despite this, in the present patient cohort, we were able to observe significant changes in CD10^low^ PMNs correlating with clinical outcome parameters. The possibility of the significant results occurring due to multiple testing should be mentioned in this context, as no corrections (such as Bonferroni's correction) were made to make potential findings in an exploratory setting more likely. Additionally, a more diverse spectrum of methods could have yielded additional insights. For example, confirming the immaturity of the CD10^low^ PMNs, for example, via fluorescence microscopy, would have strengthened the significance of the data. Despite thorough compensation control in our flow cytometric measurements (using single stains and fluorescence‐minus‐one) further confirmation using different clones and fluorochromes is needed to verify the results.

## 5 Conclusion

We discovered relevant correlations between CD10^low^ PMN numbers and established markers of inflammation and organ damage. Concerning the timing, the early changes of CD10^low^ PMN numbers appear superior to other established parameters, including CRP, IL‐6 and PCT. This implies that the analysis of CD10^low^ PMNs using (potentially automatic) flow cytometry can be used to identify patients at risk for excessive post‐surgical inflammation. This would enable physicians to make more informed diagnostic and therapeutic decisions, guiding immunomodulation and preventing negative outcomes. Future studies should prioritise confirming the identified correlations between CD10^low^ PMNs and inflammation while also investigating underlying mechanisms. A further study with more patients and a focus on the prediction of post‐surgical complications, for example, infections or organ failure, while also exploring the influence of other parameters, such as surgical techniques, would be optimal.

## Author Contributions

Conceptualization: M.H.‐L. and L.W.; Data curation: T.M.W., J.S., A.S.K. and L.W.; Formal analysis: T.M.W. and L.W.; Funding acquisition: D.A.C.M. and M.H.L; Investigation: T.M.W., J.S., A.S.K., D.J., D.A.C.M. and L.W.; Methodology: T.M.W., J.S., A.S.K., D.A.C.M. and L.W.; Project administration: M.H.‐L.; Visualisation: T.M.W. and L.W.; Writing – original draft: T.M.W. and L.W.; Writing – review and editing: all authors.

## Conflicts of Interest

The authors declare no conflicts of interest.

## Supporting information


Appendix S1.

## Data Availability

The original contributions presented in this study are included in the article/Appendix S1. The data that support the findings of this study are available from the corresponding author upon reasonable request.
