# Supplementary material for: Exploratory Study of CD10low Polymorphonuclear Leukocytes Preceding and Correlating With Postsurgical Inflammation
Source: Scand J Immunol. 2025 Jul 13;102(1):e70042. doi: 10.1111/sji.70042 (PMC12256972; doi:10.1111/sji.70042)
Supplement: Supplementary file 1 — Appendix S1. [file SJI-102-e70042-s001.docx]

**Supplementary Material**

**Supplemental Table 1:** General patient characteristics. Data illustrated as median ± interquartile range, n = 12. ICU = Intensive Care Unit.

| **Age (a)** | 62.5 (57.7 – 67.0) |
| --- | --- |
| **Sex (m : f)** | 11 : 1 |
| **ICU stay duration (d)** | 2 (2;4) |
| **Hospital stay duration (d)** | 9 (8; 12) |
| **Major comorbidities (number of patients)** | hypertension (7)  hyperlipoproteinemia (6)  diabetes mellitus type 2 (1) |

**Supplemental Table 2**: Peri-, postoperative surgical and anesthesiologic data. Data illustrated as median ± interquartile range, n = 12. ECC = extracorporeal circulation

|  | **anesthesiologic preparation (including induction)** | **surgery** | **postoperative** |
| --- | --- | --- | --- |
| **duration (min)** | 78 (61; 82) | 211 (176; 277) | shown in Supplemental Table 1 |
| **surgical duration until ECC start (min)** |  | 29 (18; 48) |  |
| **ECC total duration (min)** |  | 141 (118; 162) |  |
| **ECC ischemia duration (min)** |  | 89 (71; 109) |  |
| **ECC reperfusion duration (min)** |  | 37 (24; 42) |  |
| **Anesthesiologic medication used (number of patients)** | etomidate (12)  fentanyl (12)  heparin (12)  noradrenalin (12)  propofol (12)  sevoflurane (12)  pancuronium (9)  sufentanil (7)  rocuronium (3)  midazolam (2) |  | heparin (12)  noradrenalin (3)  propofol (11)  piritramide (8)  remifentanil (2)  sufentanil (1)  clonidine (1) |
| **Major complications (number of patients)** |  | excessive bleeding requiring red blood cell transfusion (3)  thrombocytopenia requiring platelet transfusion (2) | delirium (3)  hemothorax (1)  renal failure requiring dialysis (1)  refractory atrial fibrillation (1)  thrombocytopenia requiring platelet transfusion (1) |


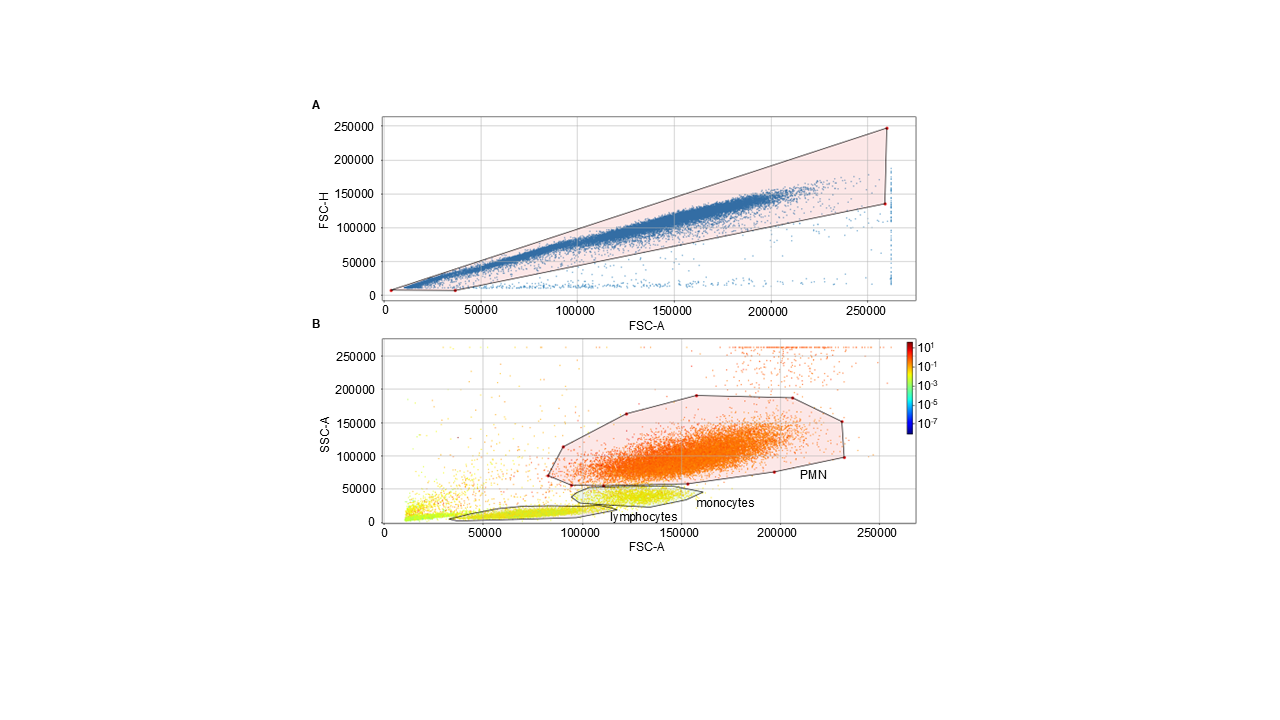


**Supplemental Figure 1**: Representative gating strategy of the flow cytometry data for polymorphonuclear leukocytes (PMNs). (**A**) Exclusion of doublets by forward scatter area (FSC-A) against FSC-height (FSC-H) to identify single particles. (**B**) Subsequent gating of PMNs by FSC-A versus side scatter area (SSC-A).


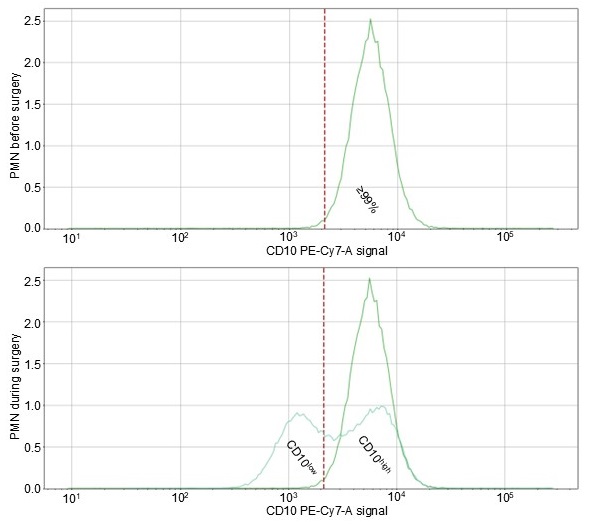


**Supplemental Figure 2**: Representative distribution of CD10 as a precursor marker for polymorphonuclear leukocytes (PMNs) from patient blood exposed to buffer control before surgery and during surgery. CD10^low^ PMNs were determined using the pre-surgery measurement time point as reference for the splitter gate to identify CD10^low^ PMNs as well as CD10^high^ PMNs.


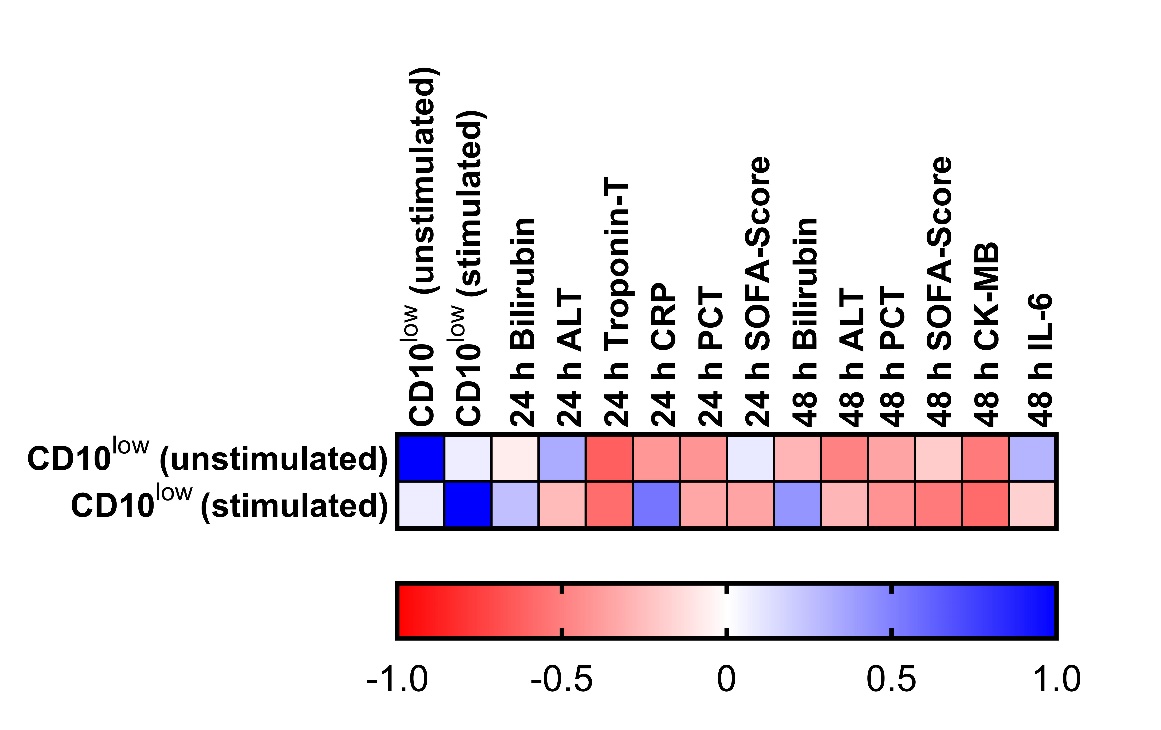


**Supplemental Figure 3:** Heatmap showing non-significant correlations between markers of inflammation and organ damage at different timepoints and the number of CD10^low^ polymorphonuclear leukocytes measured during surgery. Spearman-r values are shown color-graded, n = 10. ALT = alanine transaminase, CRP = C-reactive protein, PCT = procalcitonin, CK-MB = creatine-kinase-MB, IL-6 = interleukin-6.


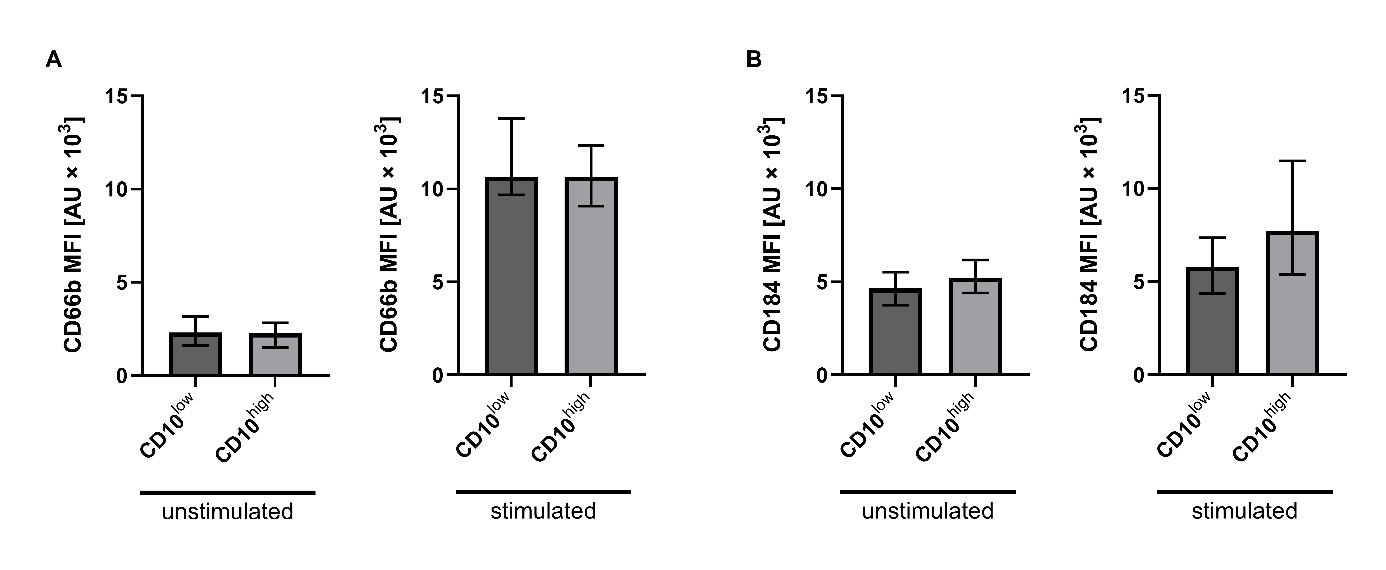


**Supplemental Figure 4**: Comparison of surface antigen expression of CD10^low^ polymorphonuclear leukocytes (PMNs) and CD10^high^ PMNs during surgery under unstimulated (with buffer as ctrl) and stimulated conditions (with a cocktail consisting of 10 µM fMLF, 1 µM PAF and 2.3 µM TNF-α). (**A**) CD66b, and (**B**) CD184 from patients during surgery. Median ± interquartile range, n=10. Mann-Whitney U Test.
